# Exogenous dietary enzyme formulations improve growth performance of broiler chickens fed a low-energy diet targeting the intestinal nutrient transporter genes

**DOI:** 10.1371/journal.pone.0198085

**Published:** 2018-05-30

**Authors:** Ahmed A. Saleh, Ali H. El-Far, Mervat A. Abdel-Latif, Mohamed A. Emam, Rania Ghanem, Hatem S. Abd El-Hamid

**Affiliations:** 1 Department of Poultry Production, Faculty of Agriculture, Kafrelsheikh University, Kafrelsheikh, Egypt; 2 Department of Biochemistry, Faculty of Veterinary Medicine, Damanhour University, Damanhour, El Beheira, Egypt; 3 Department of Nutrition and Veterinary Clinical Nutrition, Faculty of Veterinary Medicine, Damanhour University, Damanhour, El Beheira, Egypt; 4 Department of Nutrition and Veterinary Clinical Nutrition, Faculty of Veterinary Medicine, Damanhour University, Damanhour, El Beheira, Egypt; 5 Animal Health Research Institute, Mansoura Laboratory, Mansoura, Egypt; 6 Department of Poultry Diseases, Faculty of Veterinary Medicine, Damanhour University, Damanhour, El Beheira, Egypt; University of Connecticut, UNITED STATES

## Abstract

Diminishing the cost of broiler chicken diet is a critical issue in the poultry industry. Numerous studies were performed to achieve this pivotal objective by diet supplementation with alternative feed additives. In the current study, low-energy broiler rations were supplemented with different commercial multienzyme formulations to minimize the cost, and increase the digestibility and absorption of the digested macronutrients. Cobb Avian 48 broiler chicks (mixed sex, 1-d-old, *n* = 3120) were randomly allocated into six groups, and each group was subdivided into four replicates (130 birds per replicate). The birds were randomly allocated into a control group fed basal diet (CB); control group fed low-energy diet (CL); and birds fed low-energy diets supplemented with different enzyme formulations. The enzyme formulations used were Xylam 500^®^ (CLX group), Hemicell^®^ (CLH group), Avizyme^®^ (CLA group), and Megazyme^®^ (CLM group,) following the doses recommended by the manufacturers. The growth performance of CLA and CLH group birds was significantly improved when compared with CL. In comparison with CB, Avizyme^®^ significantly (*p* < 0.001) increased the intestinal *PEPT1*, *GLUT2*, *ACC*, and *IL-2* expression; PEPT1 facilitates the absorption of micronutrients. In conclusion, exogenous multienzyme complexes may be included in the low-energy diet to enhance the performance of broiler chickens (Avizyme^®^ ˃ Hemicell^®^ ˃ Megazyme^®^), and reduce the diet cost by up-regulating the expression of intestinal nutrient transporter genes, and improving the immunity and serum biochemical parameters of broiler chickens.

## Introduction

Broiler chickens are a great source of protein for human. Therefore, numerous studies focus on broiler nutrition, to maintain sustainable broiler production to meet the human demand for protein. A balanced ration formulation is hence of great importance in poultry production [1, 2].

In the poultry industry, the cost of energy-contributing ingredients constitutes about 65% of the cost of the diet. Therefore, several trials were conducted to lower the cost by reducing the percentage of some energy ingredients, along with stimulating the growth performance of broiler chickens [3]. One strategy involves enzyme supplements, which enhance such growth performance parameters as feed intake (FI), feed conversion ratio (FCR), or weight gain [4]. Enzymes are effective as supplements of cereal-based diets, e.g., wheat, barley, and corn. The content of non-starch polysaccharides (NSP) is high in such diets, and hence, the enzymes enhance the growth performance of monogastric animals [5]. FCR is improved by a dietary inclusion of exogenous enzymes in the grower phase by enhancing the digestibility and lowering the viscosity of the digesta [4, 6]. In addition, the stimulation of growth performance as a result of enzyme supplementation may be attributed to enzyme involvement in decreasing the viscosity of intestinal contents and modulation of gut microbiota [7, 8].

The inclusion of xylanase (EC 3.2.1.8) in a wheat-based diet significantly decreases heat loss, leading to greater net energy gain and a better FCR, and modulating the development of intestinal microbes in broiler chickens [9]. Olukosi et al. [10] studied the effect of protease (EC 3.4.21.62), alone or in combination with xylanase and amylase (EC 3.2.1.1), on the broiler chicken diet. The authors concluded that low doses of protease improve nutrient utilization and increase solubilization of NSP components. Such doses can also improve protein and amino acid digestibility in broilers [11], while the combination of xylanase, amylase, and protease works better than the provision of protease alone. The enzyme β-mannanase, when provided in the broiler chicken diet, hydrolyzes β-mannans, decreasing the viscosity of the intestinal content and enhancing nutrient digestibility, as well as improved intestinal environment [12].

Considering the above, the inclusion of exogenous enzymes in the broiler chicken diet aids digestibility, and leading to the generation of the building blocks of lipids, carbohydrates, and proteins (fatty acids, monosaccharides, and amino acids, respectively). Therefore, the current study was performed to assess the impact of various commercial multienzyme complexes on the growth performance and expression of intestinal nutrient transporter genes in broiler chickens.

## Material and methods

### Ethics statement

The current study was performed in the research unit of the Al-Sabeel Al-Gadidah Company for Poultry Production (Tanta, Al-Gharbia, Egypt). The study was approved by the Committee of Local Experimental Animal Care of the Faculty of Veterinary Medicine (Damanhour University, Egypt). All precautions were followed to decrease animal suffering throughout the experiment.

### Birds and their management

Cobb Avian 48 chicks (mixed sex, 1-d-old, *n* = 3120), bred in-house, were randomly allocated into six groups, with each group subdivided into four replicates (130 birds per replicate). The birds were floor-reared in same-sized pens. Birds had *ad libitum* access to feed and water, and received the experimental diets for 5 consecutive weeks. The housing temperature of 32°C was gradually decreased, reaching 26°C when the chicks were 28-d-old. The chicks were exposed to a 23-h light period.

### Feeding trials

The basal starter, grower, and finisher diets, corn/soybean based, met the recommendation of the Cobb Avian 48 brochure (2014) Nutrient for broiler chickens (Table 1). The chemical composition of the basal diet was analyzed according to AOAC [13]. The birds were randomly allocated to control groups fed the basal diet (CB); control fed low-energy diet (CL); and groups fed low-energy diet containing different enzyme formulations. The enzyme formulations used were Xylam 500^®^ (0.05% *w*/*w*; CLX diet; 8000 U/g of amylase and 1620 U/g of endo-1,4-β-xylanase, Murex Company for Feed Enzymes Production, Paris, France), Hemicell^®^ (0.033% *w*/*w*; CLH diet; endo-1,4-β-d-mannanase above 16×10^4^ U/g, Elanco Company, Greenfield, IN, USA), Avizyme^®^ [0.01% *w*/*w*; CLA diet; 5000 U/g of endo-1,4-β-xylanase and 1600 U/g of subtilisin (protease), Danisco Animal Nutrition Company, Marlborough, UK)], and Megazyme^®^ [0.1% *w*/*w*; CLM diet; 1400 U/g of endo-1,4-β-xylanase and 2000 U/g of endo-1,3(4)-β-gluconase, GRUP OMEGA Company, Spain).

**Table 1:** Composition of the experimental starter, grower, and finisher diets.

Item	Starter		Grower		Finisher	
	CB	CL	CB	CL	CB	CL
Yellow corn	528	537	582	593	638	650
Soy bean 44%	350	353	286	285	215	214
Gluten 62%	53	50	60	60	70	69
Soy bean oil	29	20	23	13	27	17
Dicalcium phosphate	17	17	16	16	16	16
dl-Methionine*	2	2	1.8	1.8	1.2	1.2
l-Lysine**	1.3	1.3	1.4	1.4	2.4	2.4
Threonine	0.5	0.5	0.3	0.3	0.1	0.1
Lime stone	11	11	11	11	10	10
NaCl	3.5	3.5	3.5	3.5	3.5	3.5
Premix***	3	3	3	3	3	3
Sodium bicarbonate	1.5	1.5	1.5	1.5	1.6	1.6
Potassium carbonate	0.2	0.2	0.5	0.5	2.2	2.2
Starch			10	10	10	10
**Calculated composition, %**						
Crude protein	23	23	21	21.1	19	19
ME (kcal/kg)	3001	2952	3051	3001	3151	3102
Calcium	0.9	0.9	0.9	0.9	0.8	0.8
Available phosphorus	0.4	0.4	0.4	0.4	0.4	0.4
Lysine	1.3	1.3	1.1	1.1	1	1
Methionine	0.6	0.6	0.6	0.6	0.5	0.5
Threonine	0.8	0.8	0.7	0.7	0.6	0.6

* 99% feed grade (Ningbo Haixin Co., Zhejiang, China).

** 99% feed grade

*** Hero mix^®^ (Hero pharm, Cairo, Egypt). Composition (per 3 kg): vitamin A, 12,000,000 IU; vitamin D3, 2,500,000 IU; vitamin E, 10,000 mg; vitamin K3, 2000 mg; vitamin B1, 1000 mg; vitamin B2, 5000 mg; vitamin B6, 1500 mg; vitamin B12, 10 mg; niacin 30,000 mg; biotin, 50 mg; folic acid, 1000 mg; pantothenic acid, 10,000 mg; manganese, 60,000 mg; zinc, 50,000 mg; iron, 30,000 mg; copper, 4000 mg; iodine, 300 mg; selenium, 100 mg; and cobalt, 100 mg.

CB, control fed basal diet; CL, control fed low-energy diet.

### Vaccination

All birds were vaccinated as follows. The Newcastle disease and avian influenza vaccines (Volvac^®^ B.E.S.T. AI+ND, Boehringer Ingelheim Co., Ingelheim am Rhein, Germany) were provided on day 7, by subcutaneous injection in the neck. On day 14, the birds were vaccinated using Nobilis^®^ GUMBORO D78 (Intervet, Netherlands) and Nobilis^®^ ND LaSota (Intervet) vaccines by eye drops following manufacturers’ recommendations.

### Sample collection

Blood samples (*n =* 20 per group) were collected from the wing vein from 14-d-old and 28-d-old birds, without anticoagulant, and centrifuged at 1435 *×g* for 5 min at 4°C. The collected clear sera were used in hemagglutination inhibition (HI) assays (section 2.7) and biochemical analyses (section 2.8).

At the end of the experiment (day 35), four birds from each replicate were sacrificed under anesthesia by intravenous injection of sodium pentobarbital (50 mg/kg) and necropsies were immediately performed. Samples (*n =* 16) of the ileum (1-cm pieces) and the Meckel’s diverticulum (5-cm pieces) were taken, and immediately washed in physiological saline (0.9% NaCl). Each sample was placed in an Eppendorf tube and instantly frozen in liquid nitrogen.

### Growth indices

Performance parameters, i.e., average body weight (BW), voluntary feed intake (VFI), body weight gain (BWG), and FCR were determined weekly throughout the entire experimental period. European production efficiency factor (EPEF) was also determined throughout the entire experimental period [14].

### Hemagglutination inhibition assay

Antibody titers for the Newcastle disease vaccine (NDV) were determined by the HI test on days 14 and 28. Briefly, two-fold serial dilutions of serum samples (0.025 ml) were prepared in normal saline in 96 wells plate. Equal volumes of the NDV antigen were added to each well of the plate [15]. Three rows of wells were the controls: the first row contained the NDV antiserum (positive control); the second row contained NDV antigen alone (negative control); and the third row contained normal saline with red blood cells (reagent control). The plate was left for 10 min at 25°C, and 0.05 ml of chicken red blood cells were added to each well. The plate was then shaken and left until a pattern of agglutination emerged. HI titers are presented as the reciprocal of the highest dilution that caused 50% agglutination inhibition; log2 titers were calculated [16].

Antibody titers for the infectious bursal disease (IBD) were determined on day 28 using the ProFLOK^®^ IBD PLUS ELISA kit (Synbiotics, Corp., Kansas City, MO, USA) [17], developed primarily to aid the detection of pre- and post-vaccination IBD antibody levels in chickens.

### Biochemical analysis

Biochemical analyses of the collected sera were performed to determine total protein, albumin, alanine aminotransferase (ALT, EC 2.6.1.2), aspartate aminotransferase (AST, EC 2.6.1.1), total cholesterol, triacylglycerol (TAG), uric acid, and creatinine levels, following the instructions enclosed in the appropriate kits of the Biodiagnostic Company (Giza, Egypt). Serum globulin levels were calculated by subtracting the albumin concentration from the total protein concentration in sample [18].

### RNA extraction and reverse-transcription polymerase chain reaction (RT-PCR)

RNA was extracted from the intestinal samples (*n =* 16 per group) using QIAamp RNeasy mini kit (Qiagen, Germany). Oligonucleotide primers were supplied by Metabion (Germany) (Tables 2 and 3).

**Table 2:** Primer sequences, target genes, and cycling conditions for SYBR green RT-PCR.

Target gene	Primer sequences (5’–3’)	Reverse transcription	Primary denaturation	Amplification (40 cycles)			Dissociation curve (1 cycle)			Product size (bp)	Accession no.	References
				Secondary denaturation	Annealing (optics on)	Extension	Secondary denaturation	Annealing	Final denaturation			
*β-actin*	F: CCACCGCAAATGCTTCTAAAC	50°C 30 min	94°C 5 min	94°C 15 s	51°C 30 s	72°C 30 s	94°C 1 min	55°C 1 min	94°C 1 min	175	NM205518	[47]
	R: AAGACTGCTGCTGACACCTTC											
*ACC*	F: AATGGCAGCTTTGGAGGTGT				60.9°C 30 s			60.9°C 1 min		119	NM_205505	[48]
	R: TCTGTTTGGGTGGGAGGTG											
*CPT1*	F: CAATGAGGTACTCCCTGAAA				57.5°C 30 s			57.5°C 1 min		337	AY675193	
	R: CATTATTGGTCCACGCCCTC											
*PEPT1*	F: CCCCTGAGGAGGATCACTGTT				60°C 30 s			60°C 1 min		205	NM_204365	[49]
	R: CAAAAGAGCAGCAGCAACGA											
*GLUT2*	F: CACACTATGGGCGCATGCT				60°C 30 s			60°C 1 min		116	NM_207178.1	
	R: ATTGTCCCTGGAGGTGTTGGTG											

*β-actin*, Beta actin; *ACC*, acetyl-CoA carboxylase; *CPT1*, carnitine acyltransferase I; *PEPT1*, peptide transporter 1; *GLUT2*, glucose transporter 2.

**Table 3:** Primer sequences, target genes, and cycling conditions for Taqman RT-PCR.

Target gene	Primer and probe sequences (5’–3’)	Reverse transcription	Primary denaturation	Amplification (40 cycles)		Exon boundary	Accession no.*	References
				Secondary denaturation	Annealing and extension (optics on)			
*28S rRNA*	F: GGCGAAGCCAGAGGAAACT	50°C 30 min.	94°C 5 min	94°C 15 s	60°C 1 min		X59733	[50]
	R: GACGACCGATTTGCACGTC							
	(FAM) AGGACCGCTACGGACCTCCACCA (TAMRA)							
*IL-2*	F: TTGGAAAATATCAAGAACAAGATTCATC				59°C 1 min	2/3	AJ009800	[51]
	R: TCCCAGGTAACACTGCAGAGTTT							
	(FAM) ACTGAGACCCAGGAGTGCACCCAGC (TAMRA)							

*IL-2*, interleukin-2

* Refers to the genomic DNA sequence.

*SYBR green RT-PCR*: PCR amplifications were performed in 25-μl reactions containing 12.5 μl of 2× QuantiTect SYBR Green PCR master mix (Qiagen), 0.25 μl of RevertAid reverse transcriptase (200 U/μl) (Thermo Fisher Scientific, Germany), 0.5 μl of each primer (20 pmol final concentration), 8.25 μl of water, and 3 μl of the RNA template. The amplifications were performed using a Stratagene MX3005P real-time PCR machine (Agilent Technologies, California, USA).

*TaqMan RT-PCR*: PCR amplifications were performed in 25-μl reactions containing 12.5 μl of 2× QuantiTect Probe RT-PCR master mix, 0.25 μl of QuantiTect RT mix, 0.5 μl of each primer (20 pmol final concentration), 0.125 μl of each probe (30 pmol concentration), 8.125 μl of PCR-grade water, and 3 μl of the RNA template. The amplifications were performed using a Stratagene MX3005P real-time PCR machine.

Stratagene MX3005P software was used to analyze the RT-PCR data, amplification curves, and cycle threshold (CT) values. Relative gene expression in different samples was evaluated by comparing the CT value of each sample with that of the positive control, following the ΔΔCt method according to Yuan et al. [19].

### Statistical analysis

Statistical analyses were performed using the SPSS program (IBM SPSS. 20^®^, IBM Corp., Armonk, NY, USA), using one-way analysis of variance (ANOVA) with Duncan’s multiple range tests. RT-PCR data were analyzed by one-way ANOVA following Tukey’s *post hoc* multiple range test in GraphPad Prism 5 (GraphPad Software, San Diego, CA, USA). Values of *p* < 0.05 were considered to signify statistically significant differences.

## Results

### Growth performance indices

The effect of the interaction of multienzyme complexes with the diet on chick growth performance after 35 d is presented in Table 4 and S1 Data. Statistical analysis of the initial BW did not reveal any significant differences between the groups.

**Table 4:** The effect of the interaction of multi-enzyme complexes and the diet on performance at 35 d.

	iBW (g)	fBW (g)	BWG (g)	VFI (g)	FCR	EPEF
**CB**	47 ± 0.1 ^a^	2104.25 ± 22 ^a^	2057.25 ± 21 ^a^	3587 ± 27 ^a^	1.705 ± 0.2 ^b^	330 ± 12 ^a^
**CL**	46 ± 0.2 ^a^	2003.25 ± 25 ^b^	1957.25 ± 24 ^b^	3411 ± 36 ^b^	1.703 ± 0.1 ^b^	321 ± 13 ^a^
**CLX**	47 ± 0.4 ^a^	2013 ± 21 ^b^	1966 ± 21 ^b^	3428 ± 28 ^b^	1.703 ± 0.3 ^b^	327 ± 13 ^a^
**CLH**	47 ± 0.4 ^a^	2010.75 ± 23 ^b^	1963.75 ± 22 ^b^	3447 ± 26 ^b^	1.715 ± 0.2 ^a^	319 ± 19 ^a^
**CLA**	46 ± 0.2 ^a^	2035 ± 24 ^ab^	1989 ± 24 ^ab^	3455 ± 44 ^b^	1.698 ± 0.1 ^b^	325 ± 17 ^a^
**CLM**	47 ± 0.3 ^a^	2004.25 ± 20 ^b^	1957.25 ± 19 ^b^	3433 ± 30 ^b^	1.713 ± 0.2 ^ab^	319 ± 15 ^a^

Mean values with different letters in the same column differ significantly at *p* < 0.05. Values are expressed as means **±** standard error. Data were analyzed by one-way ANOVA and Duncan’s multiple range test.

iBW, initial body weight; fBW, final body weight; BWG, body weight gain; VFI, voluntary feed intake; FCR, feed conversion ratio; EPEF, European production efficiency factor.

CB, control fed basal diet; CL, control fed low-energy diet; CLX, control fed low-energy diet containing Xylam 500^®^; CLH, control fed low-energy diet containing Hemicell^®^; CLA, control fed low-energy diet containing Avizyme^®^; CLM, control fed low-energy diet containing Megazyme^®^.

No significant differences in the final BW of birds in the CB and CLA groups were noted, while the final BW of animals in the CL and other enzyme-supplemented groups were significantly lower than those of animals in the CB group.

Except for the CLA group, the BWG in all low-energy diet groups supplemented or not with different enzyme complexes, were lower than those of the CB group birds. No significant difference of BWG between the CLA and CB group birds were observed.

FI of the CL and all enzyme-supplemented groups was lower than that of the CB group. Moreover, compared with the CB group, inclusion of the different enzyme mixtures in the low-energy diet groups did not significantly improve the FCR. In contrast, FCR was impaired in the CLH group. No significant difference in the EPEF was observed between groups.

### Hemagglutination inhibition assay outcomes

As shown in Table 5, no significant changes from the CB group in the ND antibody titers of the CLH and CLA group animals were observed on day 28. In contrast, the ND titers were significantly lower in the CL, CLX, and CLM groups than that in the CB group. This indicated that some of the provided enzyme complexes did not interfere with the high ND titers even in birds fed low-energy diet.

**Table 5:** The effect of the interaction of multi-enzyme complexes and the diet on antibody titers (log2) against ND and IBD.

	ND		IBD
	Day 14	Day 28	Day 28
**CB**	5.66 ± 0.2 ^a^	2.66 ± 0.10 ^a^	4.16 ± 0.2 ^ab^
**CL**	5.66 ± 0.3 ^a^	1.66 ± 0.10 ^b^	3.83 ± 0.3 ^b^
**CLX**	6.66 ± 0.4 ^a^	1.66 ± 0.13 ^b^	2.83 ± 0.4 ^c^
**CLH**	5.83 ± 0.6 ^a^	3.00 ± 0.30 ^a^	4.83 ± 0.3 ^a^
**CLA**	6.00 ± 0.4 ^a^	2.50 ± 0.20 ^a^	4.50 ± 0.4 ^a^
**CLM**	5.66 ± 0.4 ^a^	1.83 ± 0.40 ^b^	4.33 ± 0.4 ^a^

Mean values with different letters in the same column differ significantly at *p* < 0.05. Values are expressed as means **±** standard error. Data were analyzed by one-way ANOVA and Duncan’s multiple range test.

CB, control fed basal diet; CL, control fed low-energy diet; CLX, control fed low-energy diet containing Xylam 500^®^; CLH, control fed low-energy diet containing Hemicell^®^; CLA, control fed low-energy diet containing Avizyme^®^; CLM, control fed low-energy diet containing Megazyme^®^.

Regarding the IBD antibody titers on day 28, no significant changes in birds from the CLH, CLA, and CLM groups were apparent in comparison with the CB group animals. In contrast, the IBD antibody titers in the CLX group were significantly lower than in animals from all other groups (S1 Data).

### Biochemical assay outcomes

As shown in Table 6, no significant changes (*p* ˃ 0.05) in total protein and albumin levels in the CLX, CLH, and CLA groups were apparent, while they were significantly lower in the CLM group (*p* < 0.05). No significant differences of globulin, creatinine, ALT, and AST levels were noted in birds from the CB, CL, and enzyme-supplemented groups. Serum uric acid levels were significantly lower (*p* < 0.05) in the CLX, CLH, and CLA groups than those in the CB group.

**Table 6:** The effect of the interaction of multi-enzyme complexes and the diet on serum parameters in broiler chickens.

	CB	CL	CLX	CLH	CLA	CLM
**Total protein (g/dl) **	5.86 ± 0.31 ^a^	6.20 ± 0.92 ^a^	4.83 ± 0.40 ^ab^	5.20 ± 0.41 ^ab^	4.73 ± 0.51 ^ab^	3.78 ± 0.16 ^b^
**Albumin (g/dl) **	4.27 ± 0.25 ^a^	4.33 ± 0.81 ^a^	2.99 ± 0.27 ^ab^	3.26 ± 0.24 ^ab^	2.96 ± 0.44 ^ab^	2.25 ± 0.11^b^
**Globulin (g/dl) **	1.59 ± 0.07 ^a^	1.87 ± 0.14 ^a^	1.84 ± 0.14 ^a^	1.94 ± 0.19 ^a^	1.77 ± 0.11 ^a^	1.53 ± 0.08 ^a^
**ALT (U/L)**	5.98 ± 0.58 ^a^	5.04 ± 0.69 ^a^	4.89 ± 1.16 ^a^	5.67 ± 1.09 ^a^	6.11 ± 0.87 ^a^	6.11 ± 0.87 ^a^
**AST (U/L)**	251.80 ± 6.22 ^a^	240.51 ± 4.48 ^a^	239.43 ± 3.26 ^a^	245.54 ± 7.06 ^a^	233.47 ± 6.33 ^a^	240.30 ± 6.50 ^a^
**Total cholesterol (mg/dl)**	129.71 ± 6.24 ^ab^	149.72 ± 9.63 ^ab^	153.22 ± 9.1 ^a^	145.16 ± 8.95 ^ab^	122.87 ± 6.56 ^b^	138.45 ± 9.67 ^ab^
**TAG (mg/dl)**	114.50 ± 2.83 ^ab^	94.32 ± 8.53 ^c^	101.42 ± 5.22 ^bc^	101.20 ± 5.21^bc^	122.70 ± 6.16 ^a^	92.37 ± 5.13^c^
**Uric acid (mg/dl)**	7.33 ± 0.58 ^a^	7.03 ± 0.12 ^ab^	6.43 ± 0.22 ^bc^	6.13 ± 0.23 ^c^	6.31 ± 0.14 ^c^	6.43 ± 0.14 ^bc^
**Creatinine (mg/dl)**	1.06 ± 0.17 ^a^	0.90 ± 0.09 ^a^	0.89 ± 0.02 ^a^	0.81 ± 0.05 ^a^	0.97 ± 0.21 ^a^	0.90 ± 0.08 ^a^

Mean values with different letters in the same row differ significantly at *p* < 0.05. Values are expressed as means **±** standard error. Data were analyzed by one-way ANOVA and Duncan’s multiple range test.

CB, control fed basal diet; CL, control fed low-energy diet; CLX, control fed low-energy diet containing Xylam 500^®^; CLH, control fed low-energy diet containing Hemicell^®^; CLA, control fed low-energy diet containing Avizyme^®^; CLM, control fed low-energy diet containing Megazyme^®^.

### Gene expression analysis

**Fig 1:**
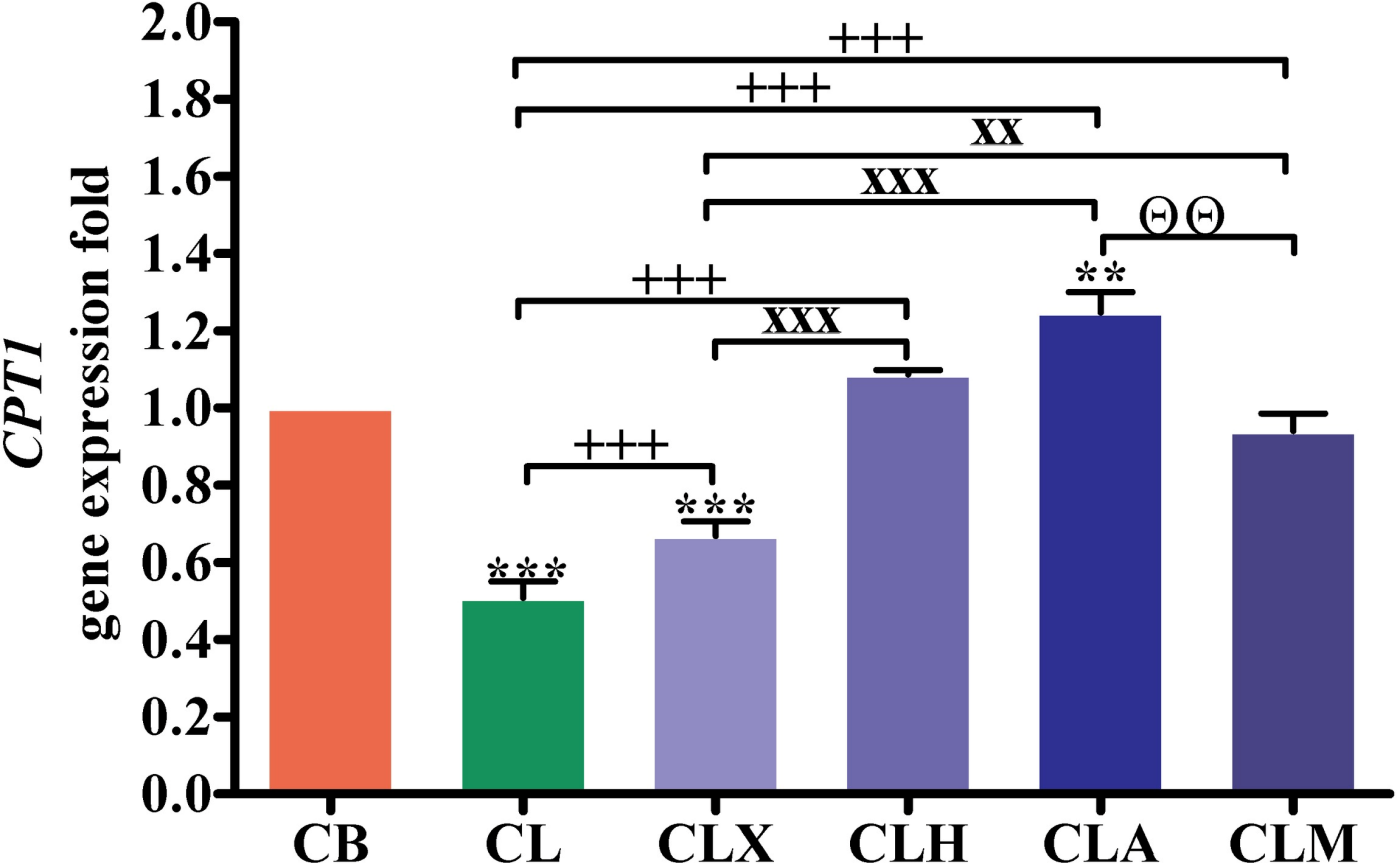
RT-PCR analysis of the *CPT1* gene expression. Gene expression was analyzed in intestinal samples (*n* = 16). **p < 0.01 and ***p < 0.001 vs. CB. ^+++^*p* < 0.001 vs. CL. ^xx^*p* < 0.01 and ^xxx^*p* < 0.001 vs. CLX. ^ΘΘ^*p* < 0.01 vs. CLA. Statistical analysis was performed using one-way ANOVA and Tukey’s *post hoc* test for multiple comparisons. CB, control fed basal diet; CL, control fed low-energy diet; CLX, control fed low-energy diet containing Xylam 500^®^; CLH, control fed low-energy diet containing Hemicell^®^; CLA, control fed low-energy diet containing Avizyme^®^; CLM, control fed low-energy diet containing Megazyme^®^.

As shown in Fig 1 and supplemented in S1 Data, the expression of the intestinal carnitine acyltransferase I gene (*CPT1*) in CL and CLX groups was significantly lower (*p* < 0.001) than that in the CB group, while it was significantly higher (*p* < 0.01) in the CLA group. *CPT1* expression was significantly higher (*p* < 0.001) in the CLX, CLH, CLA, and CLM groups than that in the CL group. It was also significantly higher (*p* < 0.001) in the CLA and CLH groups, and in the CLM group (*p* < 0.01), than that in the CLX group. Moreover, *CPT1* gene expression in birds from the CLA groups was significantly higher (*p* < 0.01) than that in the CLM group.

**Fig 2:**
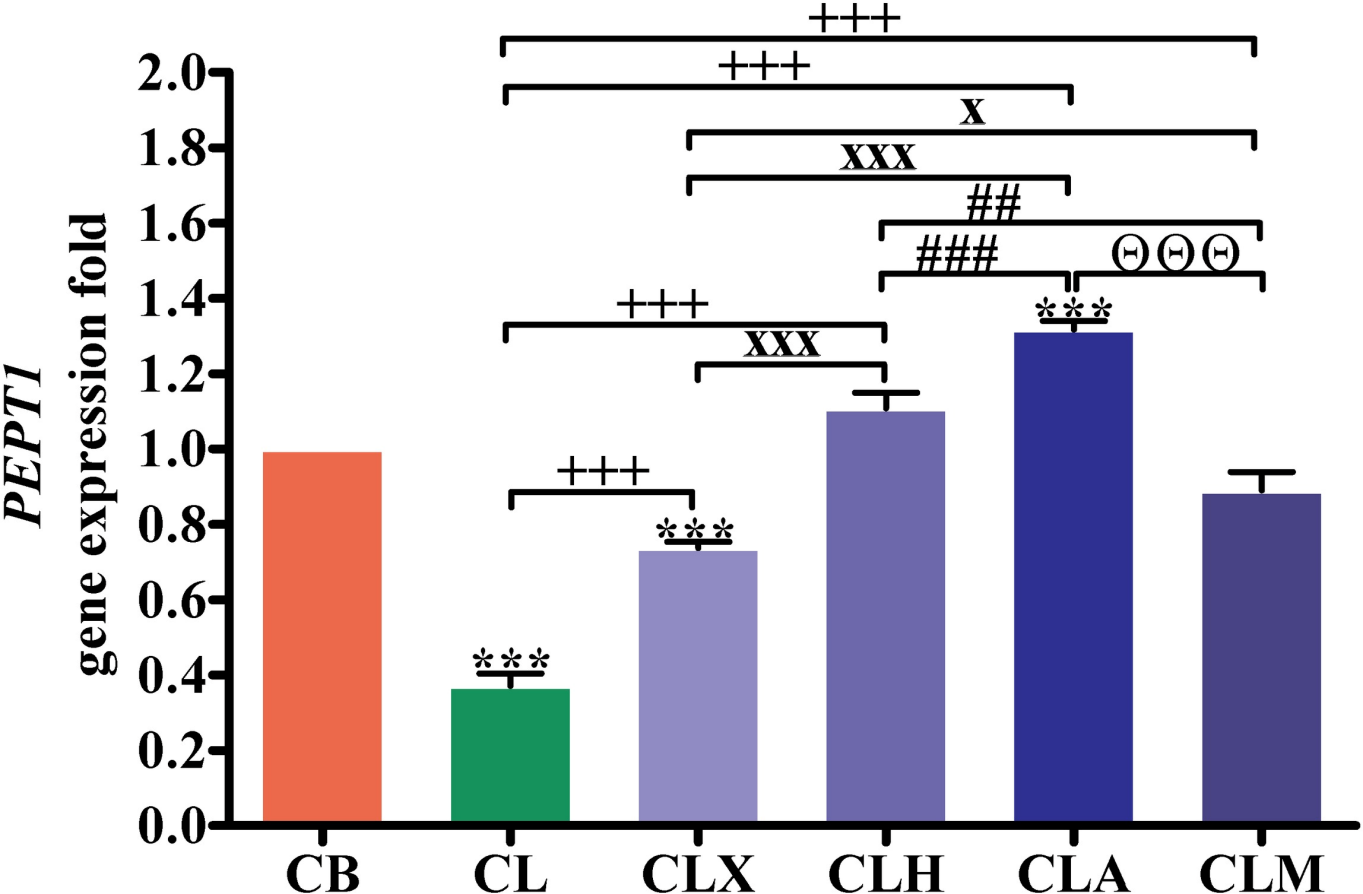
RT-PCR validation of the *PEPT1* gene. Gene expression was analyzed in intestinal samples (*n* = 16). ***p < 0.001 vs. CB. ^+++^*p* < 0.001 vs. CL. ^x^*p* < 0.05, ^xx^*p* < 0.01, and ^xxx^*p* < 0.001 vs. CLX. ^#^*p* < 0.05, ^##^*p* < 0.01, and ^###^*p* < 0.001 vs. CLH. ^ΘΘΘ^*p* < 0.001 vs. CLA. Statistical analysis was performed using one-way ANOVA and Tukey’s *post hoc* test for multiple comparisons. CB, control fed basal diet; CL, control fed low-energy diet; CLX, control fed low-energy diet containing Xylam 500^®^; CLH, control fed low-energy diet containing Hemicell^®^; CLA, control fed low-energy diet containing Avizyme^®^; CLM, control fed low-energy diet containing Megazyme^®^.

As shown in Fig 2, the expression of the intestinal peptide transporter 1 gene (*PEPT1*) was significantly lower (*p* < 0.001) in birds from the CL and CLX groups than that in CB. In contrast, *PEPT1* expression in the CLA group was significantly higher (*p* < 0.001) than that in the CB group. In all enzyme-supplemented groups, the *PEPT1* expression was significantly higher (*p* < 0.001) than that in the CL group. The *PEPT1* expression was significantly higher in the CLA (*p* < 0.001), CLH (*p* < 0.001), and CLM (*p* < 0.05) groups than that in the CLX group. Similarly, expression of this gene in the CLA (*p* < 0.001) and CLM (*p* < 0.01) groups was significantly higher than that in the CLH group. Interestingly, *PEPT1* expression was significantly higher (*p* < 0.01) in the CLA group that that in the CLM group (S1 Data).

**Fig 3:**
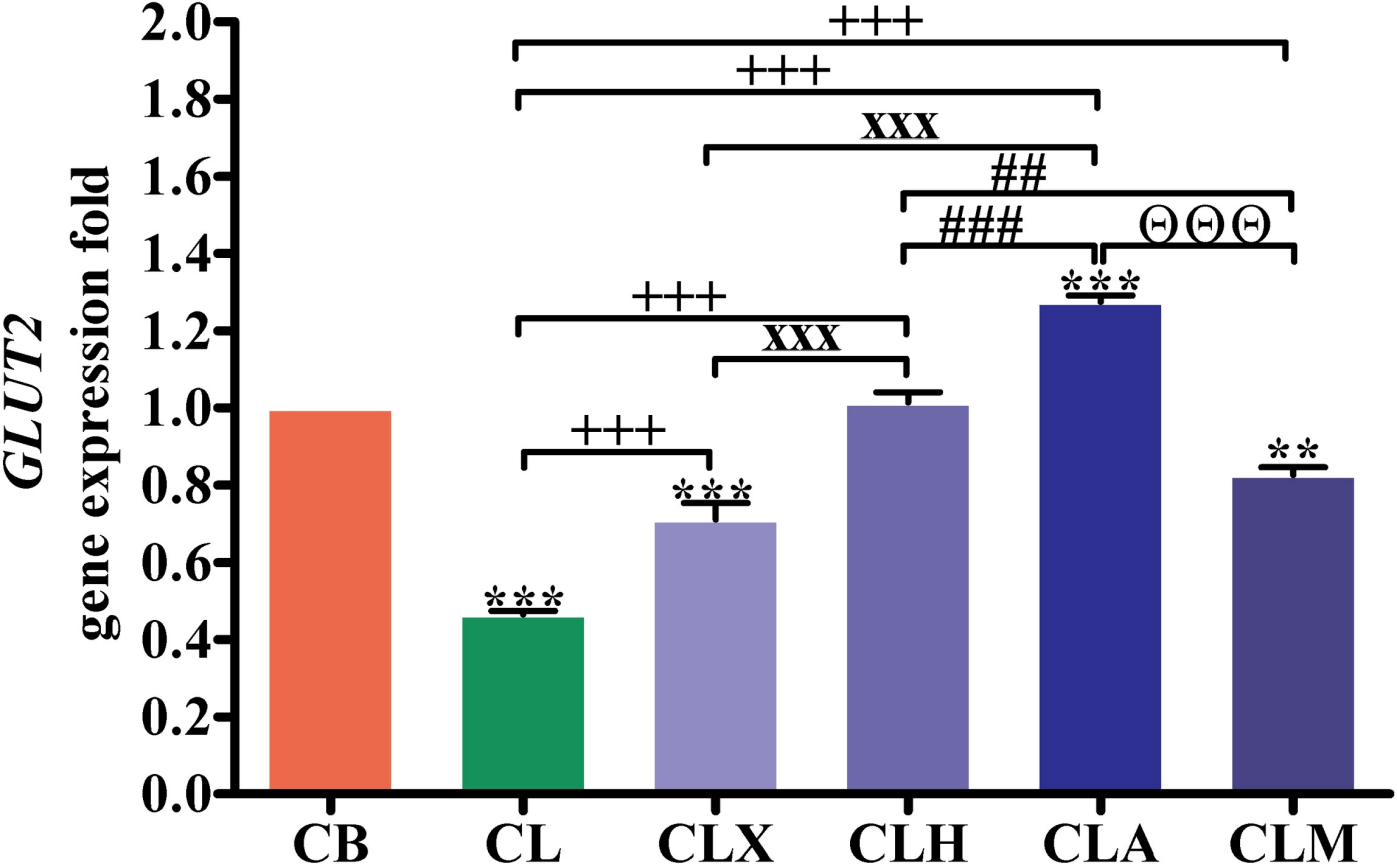
RT-PCR validation of the *GLUT2* gene. Gene expression was analyzed in intestinal samples (*n* = 16). **p < 0.01 and ***p < 0.001 vs. CB. ^+++^*p* < 0.001 vs. CL. ^xxx^*p* < 0.001 vs. CLX. ^##^*p* < 0.01 and ^###^*p* < 0.001 vs. CLH. ^Θ^*p* < 0.05, ^ΘΘ^*p* < 0.01, and ^ΘΘΘ^*p* < 0.001 vs. CLA. Statistical analysis was performed using one-way ANOVA and Tukey’s *post hoc* test for multiple comparisons. CB, control fed basal diet; CL, control fed low-energy diet; CLX, control fed low-energy diet containing Xylam 500^®^; CLH, control fed low-energy diet containing Hemicell^®^; CLA, control fed low-energy diet containing Avizyme^®^; CLM, control fed low-energy diet containing Megazyme^®^.

Fig 3 shows the expression of the intestinal glucose transporter 2 gene (*GLUT2*). The expression was low in the CLM (*p* < 0.01), and the CL and CLX (*p* < 0.001) groups, but high in the CLA group (*p* < 0.001), in comparison with that in the CB group. Compared with the CL group, *GLUT2* gene expression was significantly increased in birds from the CLA, CLH, CLM, and CLX groups (*p* < 0.001). *GLUT2* gene expression in the CLA and CLH groups was significantly higher (*p* < 0.001) than that in the CLX group. The *GLUT2* expression in birds from the CLA (*p* < 0.001) and CLM (*p* < 0.01) groups was significantly higher than that in the CLH group. Similarly, *GLUT2* gene expression in the CLA group was significantly higher (*p* < 0.001) than that in the CLM group.

**Fig 4:**
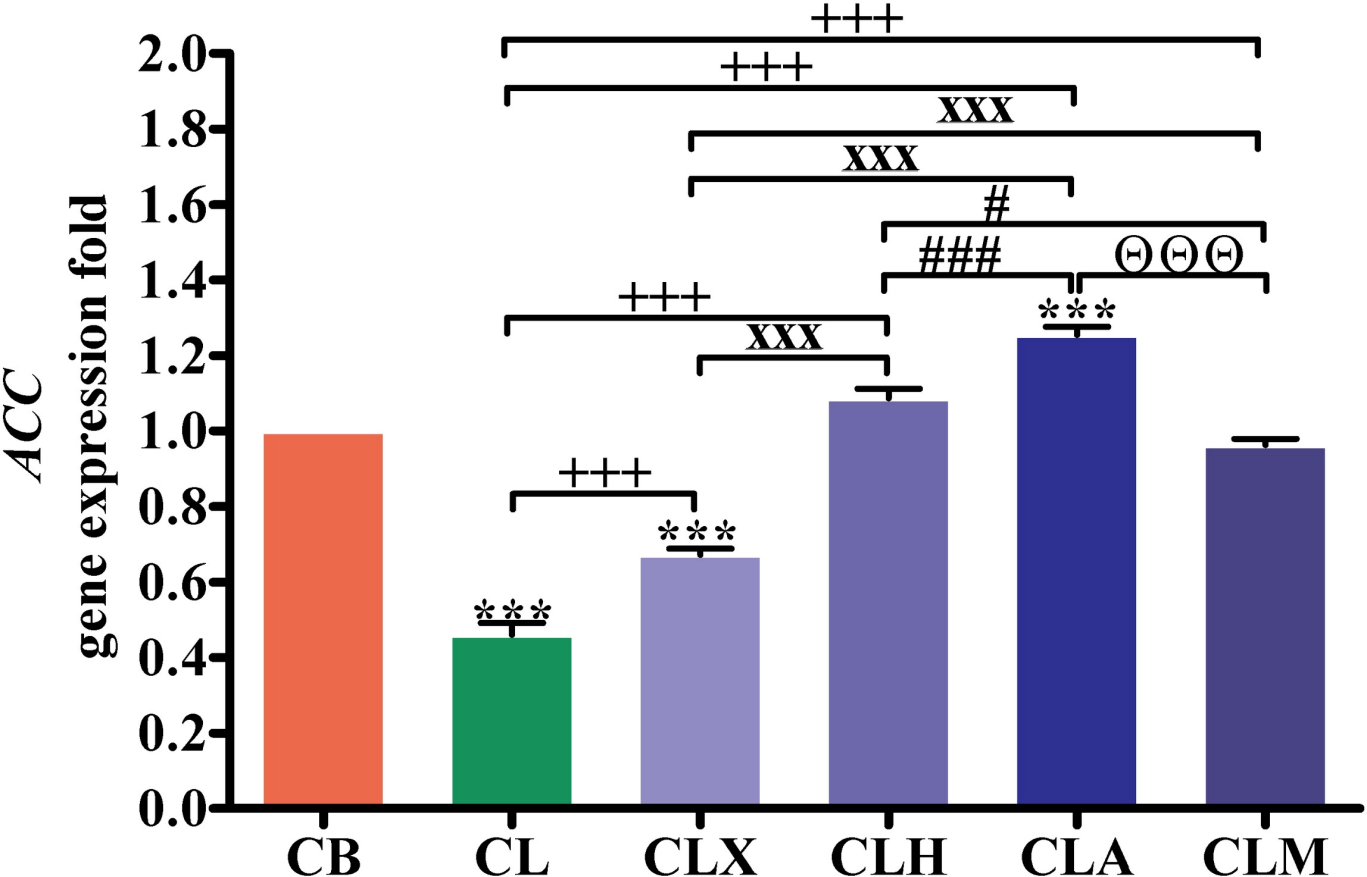
RT-PCR validation of the *ACC* gene. Gene expression was analyzed in intestinal samples (*n* = 16). ***p < 0.001 vs. CB. ^+++^*p* < 0.001 vs. CL. ^xxx^*p* < 0.001 vs. CLX. ^##^*p* < 0.01 and ^###^*p* < 0.001 vs. CLH. ^ΘΘΘ^*p* < 0.001 vs. CLA. Statistical analysis was performed using one-way ANOVA and Tukey’s *post hoc* test for multiple comparisons. CB, control fed basal diet; CL, control fed low-energy diet; CLX, control fed low-energy diet containing Xylam 500^®^; CLH, control fed low-energy diet containing Hemicell^®^; CLA, control fed low-energy diet containing Avizyme^®^; CLM, control fed low-energy diet containing Megazyme^®^.

Intestinal mRNA levels of the acetyl-CoA carboxylase gene (*ACC*) (Fig 4 and S1 Data) were significantly lower (*p* < 0.001) in the CL and CLX groups than in the CB group. *ACC* expression in all enzyme-supplemented groups was significantly higher (*p* < 0.001) than in the CL group. The expression of *ACC* gene was significantly higher (*p* < 0.001) in the CLA, CLH, and CLM groups than that in the CLX group. The *ACC* expression in the CLA group was significantly higher (*p* < 0.001) than that in the CLH group. Further, the *ACC* gene expression in the CLA group was significantly higher (*p* < 0.001) than that in the CLM group.

**Fig 5:**
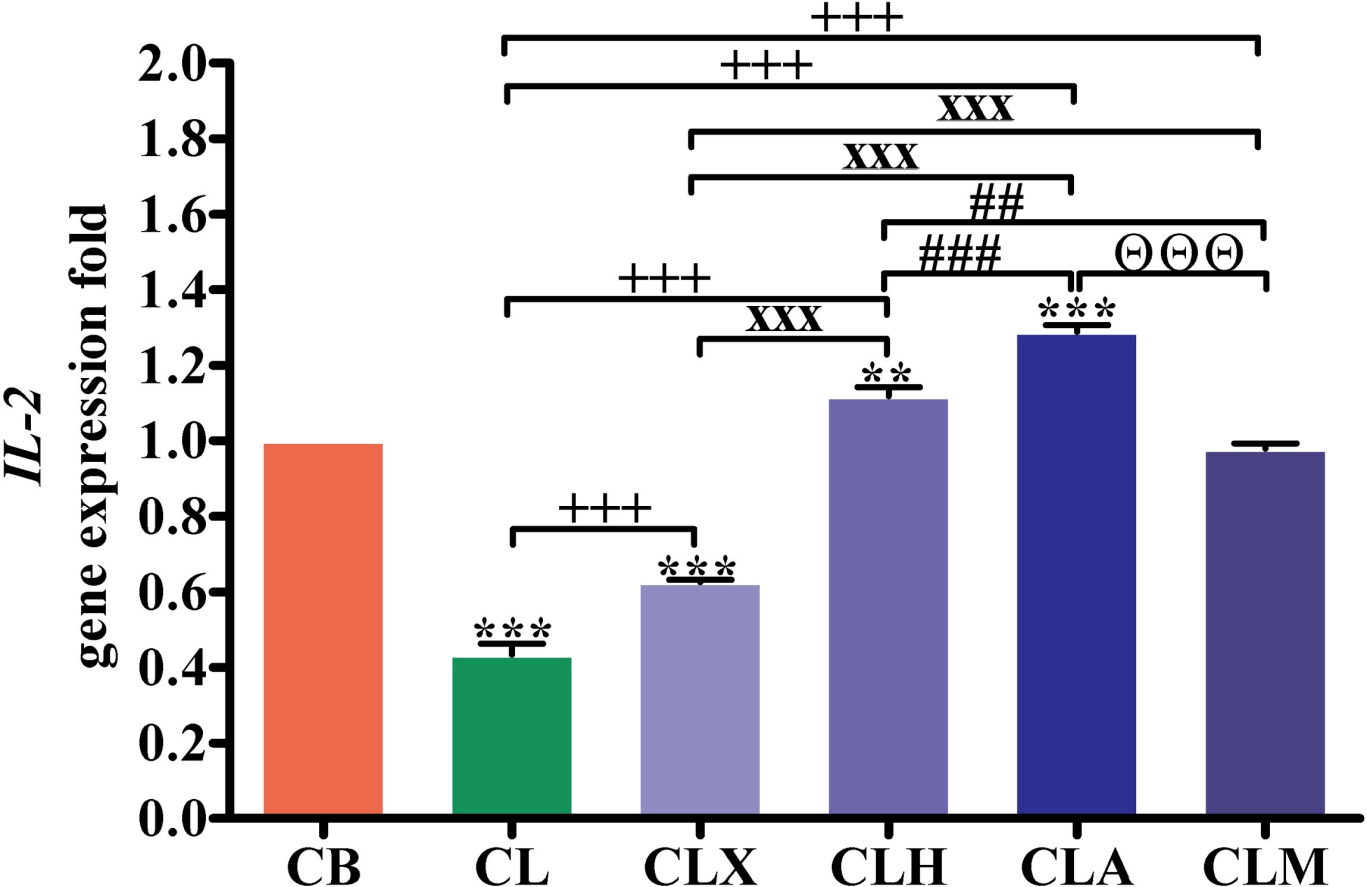
RT-PCR validation of the *IL-2* gene. Gene expression was analyzed in intestinal samples (*n* = 16). **p < 0.01 and ***p < 0.001 vs. CB. ^+++^*p* < 0.001 vs. CL. ^xxx^*p* < 0.001 vs. CLX. ^##^*p* < 0.01 and ^###^*p* < 0.001 vs. CLH. ^ΘΘΘ^*p* < 0.001 vs. CLA. Statistical analysis was performed using one-way ANOVA and Tukey’s *post hoc* test for multiple comparisons. CB, control fed basal diet; CL; control fed low-energy diet; CLX, control fed low-energy diet containing Xylam 500^®^; CLH, control fed low-energy diet containing Hemicell^®^; CLA, control fed low-energy diet containing Avizyme^®^; CLM, control fed low-energy diet containing Megazyme^®^.

The expression of the intestinal interleukin-2 (*IL-2*) gene is shown in Fig 5 and S1 Data. It was significantly lower (*p* < 0.001) in the CL and CLX birds than that in the CB, but significantly higher in the CLA (*p* < 0.001) and CLH (*p* < 0.01) groups. Relative to CL, the intestinal *IL-2* gene expression in the CLA, CLH, CLM, and CLX groups was significantly higher (*p* < 0.001) than that in the CL group. The *IL-2* gene expression was significantly higher (*p* < 0.001) in the CLA, CLH, and CLM groups than that in the CLX group. The *IL-2* gene expression in the CLA (*p* < 0.001) and CLM (*p* < 0.01) groups was significantly higher than that in the CLH group. Further, the *IL-2* gene expression in the CLA group was significantly higher (*p* < 0.001) than that in the CLM group.

## Discussion

### Growth performance indices

This study was performed to evaluate the utility of some commercially available multienzyme complexes in the most commonly used corn/soybean-based low-energy diets, i.e., diets with a reduced soy bean oil component (50 kcal/kg diet). The FCR improvement in the negative control group fed a diet supplemented with Avizyme^®^, compared with the CB group, indicated that this mixture is able to compensate for the missing dietary 50 kcal/kg by increasing the availability of xylan. This was in agreement with Café el al. [20], who reported that birds reared on diets containing Avizyme gained more net energy from the diet than control animals not fed Avizyme. Birds reared on other multienzyme mixtures had a similar FCR than the CB birds, although the FCR was significantly impaired in the CLH birds. The responses of the performance parameters to different multienzyme mixtures were consistent. It was demonstrated that arabinoxylans account for around 5.8% of corn, and are anti-nutritional factors and xylanase substrates. Inclusion of xylanase in corn-based diets improves broiler chicken performance [21, 22]. Williams et al. [23] investigated the possibility of dietary xylanase supplementation in reduced-energy diets (−66 and −132 kcal/kg), to improve broiler chicken feed utilization in comparison with birds fed energy-sufficient diets. It has been confirmed that xylanase supplementation induces a marked decrease in the viscosity of the intestinal ingesta and enhances the digestibility of nutrients in broilers [24]. The combination of xylanase, amylase, and protease in a commercial broiler diet acts on various insoluble NSP and other anti-nutritional factors commonly found in the diet, leading to the hydrolysis of indigestible bonds in the plant cell wall and indigestible protein, enabling their digestibility [25]. Freitas et al. [26] recorded an improvement in the feed-to-gain ratio in broiler chickens on a diet supplemented with protease, as well as enhanced fat and crude protein digestibility. The of enzyme complexes in conjunction with a low-energy diet is associated with economic benefits in the poultry industry (S1 Table).

### Hemagglutination inhibition and biochemical analyses

The lack of significant changes in the ND and IBD antibody titers on day 28 was accompanied by a lack of significant changes in the serum globulin levels specifically in the CLX, CLH, and CLA enzyme-supplemented groups. These enzyme complexes resulted in the ND and IBD titer levels maintained close to the CB level. This may be regarded to indicate enhanced feed digestibility of protein because of the proteases present in these enzyme complexes and up-regulated *PEPT1* gene expression that improved intestinal nutrient absorption.

The levels of liver function (ALT and AST) and kidney function (creatinine) biomarkers were not significantly different between the groups, including the enzyme-supplemented groups. The serum uric acid levels were significantly decreased in the CLH and CLX groups. Further, the serum total cholesterol and uric acid levels were significantly decreased in the CLA group. These results confirmed the general notion that feed additives should be effective and safe [27]. Further, Ahmad et al. [28] examined the impact of a dietary xylanase supplementation on serum biochemical parameters in broilers, and reported that xylanase may be safely used in poultry diet without adversely affecting vital organ function.

### Gene expression

The building units of protein, lipids, and carbohydrates are transferred by specific transporters found in the brush border of the small intestine for the absorption by enterocytes [29, 30] such as *GLUT2* that transfers monosaccharides (glucose, fructose, galactose, and mannose) across the intestinal basement membrane [31]. Di- and tri-peptides are transferred across the intestinal brush border by the product of the *PEPT1* gene [32]. Up-regulation of the transporter-encoding genes facilitates the influx of nutrient into the intestinal epithelial cells, and then to the body, by increasing nutrient transport capacity [33]. Exogenous enzyme supplements enhance the broiler chicken’s digestibility by accelerating the activity of digestive enzymes through increased substrate availability. Consequently, enhanced molecular synthesis of intestinal transporters of the lipid, carbohydrate, and protein building blocks facilitates their absorption [4].

Up-regulation of the expression of the intestinal *CPT1* gene was observed in birds fed diets supplemented with exoenzymes (CLA ˃ CLH ˃ CLM). *CPT1* encodes a mitochondrial enzyme that is responsible for the synthesis of acyl-carnitine by facilitating the transfer of the acyl group from coenzyme A (long-chain fatty acyl-CoA) to l-carnitine, which is subsequently transported to the mitochondrial matrix for energy production via β-oxidation, with the energy stored as ATP [34, 35]. Hence, exogenous enzymes (CLA ˃ CLH ˃ CLM) that induce the up-regulation of *CPT1* induce β-oxidation and energy production to satisfy the broiler chicken requirements.

Regarding the role of multienzymes in protein metabolism, the expression of the *PEPT1* gene was significantly up-regulated in birds from the CLA group. It is possible that Avizyme^®^ altered the viscosity and composition of the diet, which triggered the change in *PEPT1* gene expression. The *PEPT1* gene product is located in the intestinal brush border membrane and facilitates the uptake of di- and tripeptides from the lumen by enterocytes [36]. Xylanase present in Avizyme^®^ up-regulated the expression of *PEPT1* and other nutrient absorption-related genes in the intestine. This was also observed by Guo et al. [37] who reported that the inclusion of xylanase in the broiler chicken diet up-regulated the expression of the jejunal sodium-glucose cotransporter 1 (*SGLT1*) and *PEPT1* genes in broiler chickens. Similarly, xylanase supplementation up-regulated the expression of jejunal *SGLT1* and *PEPT1* genes [38].

In birds from the CLA group, expression of the intestinal *GLUT2* gene was up-regulated. GLUT2 facilitates the influx of glucose, fructose, galactose, and mannose through the intestinal basement membrane, and then to the liver. In the liver, all monosaccharides are converted into glucose that is released into the bloodstream, and then distributed throughout the body and utilized. Glucose enters glycolysis and the Krebs’s cycle to satisfy the body’s energy needs and to create energy reserve. Lu et al. [39] evaluated the growth-promoting effect of xylanase and live yeast supplements in growing pigs. The authors observed that on day 15 of the experiment, *GLUT2* mRNA levels were higher in the live yeast plus xylanase groups than in the control. Also, the expression of *GLUT2* and *SGLT1* genes was reportedly up-regulated after xylanase supplementation on weeks 2 and 3, respectively, since the beginning of the feeding experiment which might suggest an increased absorption capacity in birds [40].

*ACC* encodes a biotin-dependent enzyme that plays a key role in the biosynthesis of fatty acids by catalyzing an irreversible carboxylation of acetyl-CoA for malonyl-CoA production [41]. Therefore, in the current study, the significantly increased *ACC* gene expression in the CLA group suggested a lipogenic effect of Avizyme^®^ even in combination with a low-energy diet. This effect was associated with the liberation of macronutrient building blocks and, hence, increased lipogenesis because of body energy sufficiency [42].

Dignass and Podolsky [43] suggested that IL-2 may alleviate the harmful effects of injury on the integrity of the intestinal epithelium. IL-2 is secreted by activated T lymphocytes and plays a major role in the replication, maturation, and differentiation of lymphocytes. In addition, IL-2 plays a vital role in mucosal immunity [44]. In the current study, intestinal *IL-2* expression was significantly lower in the CL and CLX animals, and significantly higher in the CLA animals, than that in the CLH group. This indicated the immunostimulatory effect of the feed additives Avizyme^®^ and Hemicell^®^. In contrast, Xylam 500^®^ inhibited the expression of the *IL-2* gene.

In the current study, up-regulation of the tested intestinal transporter genes was associated with diet supplementation with enzyme complexes. The xylanase-protease combination was previously shown to increase nutrient utilization in broilers [45]. In addition, Amerah et al. [46] noted synergism between xylanase, amylase, and protease, which improved the growth performance of broilers.

## Conclusions

Enzyme supplements of low-energy diets resulted in up-regulated expression of nutrient transporters (CLA ˃ CLH ˃ CLM), which improved the absorption of micronutrients and enhanced the growth performance of broiler chickens. In conclusion, the energy values of corn/soybean-based diets for broiler chickens can be improved by diet supplementation with an enzyme cocktail of xylanase and protease, offering promising economic benefits to producers.

## Supporting information

S1 Data Raw data sheets. (XLSX)

S1 Table Economic benefits of enzyme complexes supplementation in broiler’s diet. (DOCX)

## Abbreviations

ACC: Acetyl-CoA carboxylase
ALT: Alanine aminotransferase
ANOVA: Analysis of variance
AST: Aspartate aminotransferase
BW: Body weight
BWG: Body weight gain
CPT1: Carnitine acyltransferase I
EPEF: European production efficiency factor
FCR: Feed conversion ratio
FI: Feed intake
GLUT2: Glucose transporter 2
IBD: Infectious bursal disease
IL-2: Interleukin -2
NDV: Newcastle disease vaccine
NSP: Non-starch polysaccharides
PEPT1: Peptide transporter 1
RT-PCR: Reverse-transcription polymerase chain reaction
TAG: Triacylglycerol
