# Supplementary material for: Exogenous dietary enzyme formulations improve growth performance of broiler chickens fed a low-energy diet targeting the intestinal nutrient transporter genes
Source: PLoS One. 2018 May 30;13(5):e0198085. doi: 10.1371/journal.pone.0198085 (PMC5976200; doi:10.1371/journal.pone.0198085)
Supplement: S1 Table — (DOCX) [file pone.0198085.s002.docx]

S1 Table. Economic benefits of enzyme complexes supplementation in broiler’s diet.

| **Items** | **Price/Unit**  **(LE)** | **Starter** | | **Grower** | | **Finisher** | |
| --- | --- | --- | --- | --- | --- | --- | --- |
|  |  | CB | CL | CB | CL | CB | CL |
| Yellow corn | 3.5 | 528 | 537 | 582 | 593 | 638 | 650 |
| Soy bean 44% | 7 | 350 | 353 | 286 | 285 | 215 | 214 |
| Gluten 62% | 13 | 53 | 50 | 60 | 60 | 70 | 69 |
| Soy bean oil | 17 | 29 | 20 | 23 | 13 | 27 | 17 |
| **Cost difference (LE)** |  | **139.5** | | **138.5** | | **148** | |
| ***Cost difference between all enzyme supplemented groups and CB (LE)** |  | **109.5** | | **108.5** | | **118** | |

*Enzymes cost/ton diet=30 LE.

Feed cost/Kg diet (LE)= 7 for CB, 6.858 for CL and 6.888 for all enzyme supplemented groups.

|  | **iBW (g)** | **fBW (g)** | **BWG (g)** | **VFI (g)** | **FCR** | **EPEF** |
| --- | --- | --- | --- | --- | --- | --- |
| **CB** | 47 ± 0.1 ^a^ | 2104.25 ± 22^a^ | 2057.25 ± 21^a^ | 3587 | 1.705 ± 0.2 ^b^ | 330 ± 12 ^a^ |
| **CL** | 46 ± 0.2 ^a^ | 2003.25 ± 25 ^b^ | 1957.25 ± 24 ^b^ | 3411 | 1.703 ± 0.1 ^b^ | 321 ± 13 ^a^ |
| **CLX** | 47 ± 0.4 ^a^ | 2013 ± 21 ^b^ | 1966 ± 21 ^b^ | 3428 | 1.703 ± 0.3 ^b^ | 327 ± 13 ^a^ |
| **CLH** | 47 ± 0.4 ^a^ | 2010.75 ± 23 ^b^ | 1963.75 ± 22 ^b^ | 3447 | 1.715 ± 0.2 ^a^ | 319 ± 19 ^a^ |
| **CLA** | 46 ± 0.2 ^a^ | 2035 ± 24 ^ab^ | 1989 ± 24 ^ab^ | 3455 | 1.698 ± 0.1 ^b^ | 325 ± 17 ^a^ |
| **CLM** | 47 ± 0.3 ^a^ | 2004.25 ± 20 ^b^ | 1957.25 ± 19 ^b^ | 3433 | 1.713 ± 0.2 ^ab^ | 319 ± 15 ^a^ |

| **Item** | **FCR** | **Feed cost/Kg gain** |
| --- | --- | --- |
| **CB** | **1.705 X 7** | **11.935** |
| **CL** | **1.703 X 6.858** | **11.679** |
| **CLX** | **1.703 X 6.888** | **11.730** |
| **CLH** | **1.715 X6.888** | **11.812** |
| **CLA** | **1.698 X6.888** | **11.695** |
| **CLM** | **1.713 X6.888** | **11.799** |
